# Electroencephalogram Emotion Recognition Based on 3D Feature Fusion and Convolutional Autoencoder

**DOI:** 10.3389/fncom.2021.743426

**Published:** 2021-10-18

**Authors:** Yanling An, Shaohai Hu, Xiaoying Duan, Ling Zhao, Caiyun Xie, Yingying Zhao

**Affiliations:** ^1^Institute of Information Science, Beijing Jiaotong University, Beijing, China; ^2^School of Economics and Management, Northwest University, Xi’an, China; ^3^College of Quality and Technical Supervision, Hebei University, Baoding, China; ^4^College of Electronic and Information Engineering, Hebei University, Baoding, China; ^5^Machine Vision Technology Creation Center of Hebei Province, Baoding, China

**Keywords:** emotion recognition, differential entropy, feature fusion, convolution neural network, stacked autoencoder

## Abstract

As one of the key technologies of emotion computing, emotion recognition has received great attention. Electroencephalogram (EEG) signals are spontaneous and difficult to camouflage, so they are used for emotion recognition in academic and industrial circles. In order to overcome the disadvantage that traditional machine learning based emotion recognition technology relies too much on a manual feature extraction, we propose an EEG emotion recognition algorithm based on 3D feature fusion and convolutional autoencoder (CAE). First, the differential entropy (DE) features of different frequency bands of EEG signals are fused to construct the 3D features of EEG signals, which retain the spatial information between channels. Then, the constructed 3D features are input into the CAE constructed in this paper for emotion recognition. In this paper, many experiments are carried out on the open DEAP dataset, and the recognition accuracy of valence and arousal dimensions are 89.49 and 90.76%, respectively. Therefore, the proposed method is suitable for emotion recognition tasks.

## Introduction

From the changing of physiology or psychology caused by the influence of the surrounding environment, we can know the emotions of people. Emotion can seriously affect people’s cognitive, communication, and decision-making skills. Emotion is a comprehensive process of affection from occurrence to end. It will wake up or weaken in a very short time with a change of the surrounding environment or its own needs. The complex neural mechanism of the brain can produce stress and temporary emotions (Joy et al., 2021). Emotion is a necessary condition of human social activities, which has an important impact on human daily life. Human beings reflect emotional state through physiological signals and audio-visual state. As a tool for emotion recognition, audio-visual states such as behavior, voice, intonation, eyes, and facial expressions are easily controlled by individuals and lack authenticity. Physiological signals generated spontaneously by the human body, such as electromyographic (EMG) signals, functionality near infrared spectroscopy imaging system (fNIS) signals, electrocardiogram (ECG) signals, and electroencephalogram (EEG) signals, can capture the real emotional state of humans more effectively and accurately. Among all the physiological signals, EEG signals can directly react to the changes of the human brain activity state with the changes of the emotional state with non-invasive techniques, so they have been widely concerned. In addition, since the occurrence time of emotion is often between ten to hundreds of milliseconds, the high temporal resolution of EEG makes it very suitable for capturing such fast, dynamic, and sequential cognitive events (Xin et al., 2021).

**FIGURE 1:**
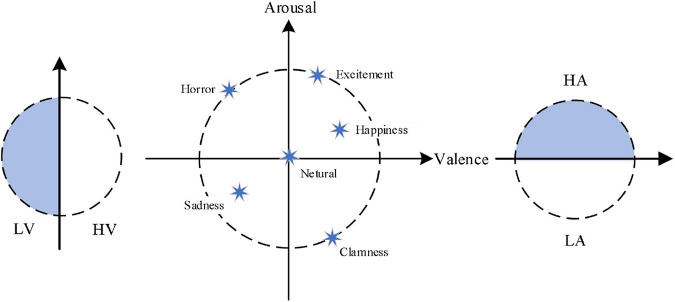
The two dimensions emotion model.

At present, in the field of emotion recognition, the emotion models can be divided into the discrete model and the dimension model. In the discrete model, each emotional state is taken as an independent emotional category. There are six basic emotions such as sadness, joy, surprise, disgust, and fear in the most widely used discrete model now. In the dimension model, the value of different dimensions of emotion quantitatively expresses each specific emotion in different positions of a coordinate system. The coordinate system is two dimensions (valence and arousal) or three dimensions (valence, arousal, and dominance). The representative theory of emotion in the two dimension classification model is Russell’s circular emotion classification model (Russell, 1980), also known as V/A (valence/arousal) emotion model, which is shown in Figure 1. In Figure 1, LV is defined as low-valence, HV is defined as high-valence, LA is defined as low-arousal, and HA is defined as high-arousal.

Recently, wearable and portable new EEG equipment has gradually entered into our horizon, and emotion recognition has also become one of the key technologies in the field of human-computer interaction today. Based on the advantage of EEG, the emotion recognition method based on EEG has aroused wide interest of scholars. Many effective emotion recognition algorithms have been proposed by scholars all over the world. For example, Guo et al. (2017) used time-frequency analysis in discrete wavelet domain for EEG feature extraction. They proposed an emotion classification based on the support vector machine (SVM) and the hidden Markov model (HMM). The new method can improve the accuracy of emotion recognition. Zheng et al. (2019) reviewed the mainstream emotion recognition algorithms based on EEG on SEED dataset and DEAP dataset. We can find that the differential entropy feature can extract the features of an EEG signal most effectively from the experimental results, and the power spectral density feature also can extract the features of the EEG signal adequately. By combining deep belief network (DBN) and entropy estimation, in Chen et al. (2018), proposed a new emotion recognition algorithm, which achieved 83.34% accuracy in the recognition of four emotions including joy, calm, sadness, and fear. In Lu et al. (2020), gave a new emotion recognition method based on dynamic entropy. In this algorithm, the best accuracy is 85.11%. Zheng (2017) presented an EEG emotion recognition algorithm based on group sparse canonical correlation analysis (GSCCA), which can improve the accuracy of emotion recognition. In Xing et al. (2019), gave a multichannel EEG emotion recognition algorithm by combining long short-term memory RNN (LSTM RNN) and stack autoencoder (SAE). In a DEAP dataset, the average accuracy of this method can achieve 81.10%. In Yin et al. (2017), used the transfer recursive feature elimination method to distinguish the state of emotion. In Liu et al. (2020), applied multi-level features guided capsule network (MLF-CapsNet) to distinguish the states of emotion, which achieved good recognition results on DEAP datasets. Tang et al. (2017) used dual-mode noise reduction automatic encoder and dual-mode long short-term memory network to identify emotional states, and they achieved 83.25% recognition accuracy on DEAP dataset. By using the Pearson correlation coefficient to rearrange EEG signals, in Wen et al. (2017), gave an emotion recognition method based on the convolution neural network (CNN), and this method achieved good results. And in Luo et al. (2020), by using spiking neural networks, Luo et al. gave a new emotion recognition method. In the DEAP dataset, this algorithm can achieve 74, 78, 80, and 86.27% in recognition accuracy of four states of arousal, valence, dominance, and preference, respectively. In Shen et al. (2021), applied multi-scale frequency bands ensemble learning to identify emotional state and achieved average recognition accuracy of 74.22% in the DEAP dataset.

In different emotion recognition algorithms, feature extraction and classification are needed whether based on traditional machine learning or deep learning. The emotion recognition algorithm based on traditional machine learning mainly relies on manual feature extraction, while the algorithm based on deep learning can realize automatic feature extraction. For emotion recognition, the research shows that the recognition rate of the method based on automatic feature extraction is higher than that based on manual feature extraction. Since the EEG signals have characteristics of non-linear and high-dimensional, it is not easy to distinguish EEG signals with a linear algorithm. Deep learning can realize end-to-end mapping, which is helpful to solve non-linear problems. In deep learning, as a typical application tool, CNN can map the data input to the output labels by automatic learning, so as to be suitable for automatic feature extraction of high-dimensional data. However, in the training stage, to achieve better performance, CNN should be fed by a lot of annotation data, which not only requires a lot of time and energy, but also easily causes over fitting. Autoencoder is composed of an encoder and a decoder and can overcome the above disadvantages. In an autoencoder, an encoder can turn source data to a hidden layer, while a decoder can map the hidden layer to source data. In addition, the dimension of hidden layer is lower than the dimension of the original feature. For a different application, encoder and decoder can be constructed by many different types of deep learning models. So, it is natural to think that CNN can be used to construct encoder and decoder networks. Therefore, in this paper, we combine CNN and autoencoder to identify emotions.

**FIGURE 2:**
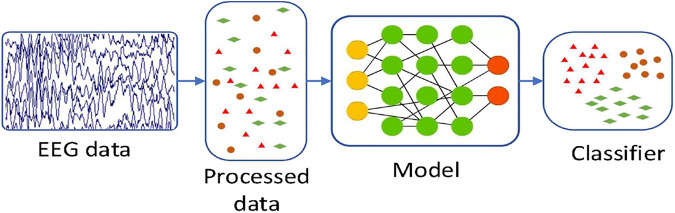
The basic process of this algorithm.

In this paper, by combining with CAE, we propose a 3D feature fusion-based emotion recognition algorithm. First, the differential entropy (DE) features of different frequency bands of EEG signals are fused to construct the 3D features of EEG signals, which retain the spatial information between channels. Then, CAE is constructed by combining CNN and stacked autoencoder (SAE) for emotion recognition. Compared with other algorithms, the proposed algorithm makes full use of the characteristics of CNN convolution layer and SAE and is suitable for an emotion recognition task. The experimental results show that CAE can further improve the recognition accuracy. Figure 2 is the basic process of the proposed algorithm in our paper. First, we use a preprocessing method to make the original data available. And then we use a 3D feature fusion to extract the features. Finally, we use CAE to recognize the state of an emotion.

The remaining parts of this paper are organized as follows: the second section introduces the basic knowledge of differential entropy, convolution neural network and stacked autoencoder; the third section introduces the algorithm flow; the fourth section introduces the dataset, experimental setup, and experimental results; the last section is the summary of this paper.

## Materials and Methods

### Differential Entropy

Entropy analysis is mainly used to classify EEG signals, which is very suitable for feature construction of EEG signals. The basic principle of entropy analysis is to extract various entropies from different frequency bands of EEG signals, and these entropies are used to construct the features with the highest discrimination between two types of signals. Previous studies have shown that differential entropy is the most widely used feature in EEG emotion classification (Shi et al., 2013). Differential entropy is the entropy of continuous random variables, which is used to characterize the complexity of continuous random variables and can be defined as


(1) $$h\,\left(x\right)=-\int_{x}f_{d}\,\left(x\right)\,\log\left(f_{d}\,\left(x\right)\right)\,dx$$

where *x* denotes the EEG signal time series; and *f*_*d*_(*x*) denotes the probability density function of *x*. The time series *x* obeys Gaussian distribution *N*(μ,σ^2^). Usually, we can represent the variance of the EEG signal by using its average energy value *P*. We assume that the length of fixed time window is *N*, the differential entropy of EEG signals can be calculated by


(2) $$h\,\left(x\right)=\frac{1}{2}\,\log\left(2\,\pi \,e\,\sigma^{2}\right)=\frac{1}{2}\,\log\left(P\right)+\frac{1}{2}\,\log\left(\frac{2\,\pi \,e}{N}\right)$$

### Convolutional Neural Network

**FIGURE 3:**
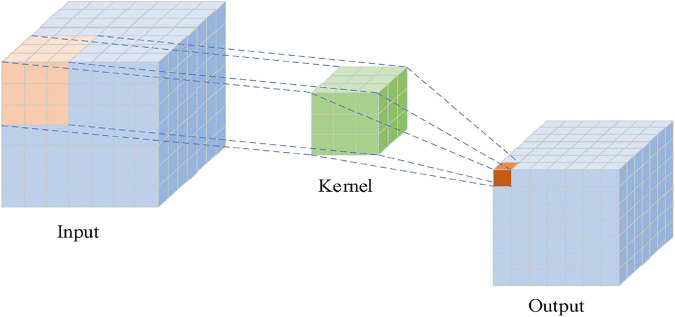
Convolution operation diagram.

In the structure of CNN, there are a convolutional layer, a pooling layer, and a fully connected layer. Convolution, which is the core of the convolution neural network, is an effective method to extract image features. The convolutional layer adopts a convolution kernel to perform the sliding convolution operation on the inputted grid data, extracting features. The distance of each sliding of the convolution kernel is called stride. Each cell of the grid data can be regarded as a neuron. The convolution kernel is essentially a weight matrix, and the so-called “convolution” operation refers to the matrix multiplication operation. Figure 3 shows the convolution operation with a 3 × 3 convolution kernel. The convolution kernel performs matrix multiplication with the input data from left to right and from top to bottom.

Traditional CNN is mostly used to extract features of 2D images. Although the collected EEG data is one-dimensional data, it can be expanded to a 3D structure to retain the spatial structure of the brain. 2D CNN will lose the original data information of EEG signals and reduce the accuracy in classification. In this paper, instead of a 2D convolution kernel, we use a 3D convolution kernel to extract the spatial features of the EEG data. Let $v_{i\,j}^{x\,y\,z}$ denote the neuron as the output value of the *j*-th features map of the *i*-th layer in (*x*,*y*,*z*), that is,


(3) $$v_{i\,j}^{x\,y\,z}=f\,\left(b_{i\,j}+\sum_{n}\sum_{p=0}^{P_{i}-1}\sum_{q=0}^{Q_{i}-1}\sum_{r=0}^{R_{i}-1}w_{i\,j\,n}^{p\,q\,r}\,v_{\left(i-1\right)\,n}^{\left(x+p\right)\,\left(y+q\right)\,\left(z+r\right)}\right)$$

where *n* is the index of the feature map of *i*-1 layer, and *b*_*ij*_ is the deviation of the convolution network. The length of the convolution kernel is denoted as *P*_*i*_. The width of the convolution kernel is denoted as *Q*_*i*_. The height of the convolution kernel is denoted as *R*_*i*_. Let $w_{i\,j\,n}^{p\,q\,r}$ be the value of the convolution kernel connected to the *n*-th feature map, and the non-linear activation function is denoted as *f*.

The pooling layer can reduce the size of the input matrix and the extract key features. There will be a certain loss of features after pooling. The pooling layer is not used since the data processed in this paper are relatively small, which will cause information loss if the pooling layer is still performed. In model training, it is necessary to calculate the loss function of the model to find the gradient for back propagation. In this paper, we use the cross-entropy loss function for model training, which is calculated based on Softmax. Softmax is the logistic regression model for binary classification. In this paper, we extend it to multi-classification by stacking it to the multinomial logistic regression model. It maps the output of multiple neurons to the (0, 1). Softmax transforms the final output of the network into a probabilistic form by exponential, as shown in Eq. 4.


(4) $$p_{i}=\frac{e^{z_{i}}}{\sum_{j=1}^{k}e^{z_{j}}}$$

Among them, *e*^*z*_*i*_^ is the network output index of the category *i*, and the denominator is the sum of the network output indexes of all categories. There are *k* categories, and *p*_*i*_ is the output probability of the category *i*.

In this paper, to seep up the net training, we use AdaDelta as the optimizer to calculate the loss through cross-entropy. The formula of cross-entropy loss is shown in Eq. 5.


(5) $$J=-\frac{1}{N}\,\sum_{1}^{N}\sum_{i=1}^{k}y_{i}\cdot \log\left(p_{i}\right)$$

where *y*_*i*_ can be denoted as the real label of the category *i*. Let *p*_*i*_ denote the probability value of the category *i* predicted by Softmax. In addition, the number of categories is denoted as *k*. The total number of samples is denoted as *N*.

### Stacked Autoencoder

There are input layer, hidden layer, and output layer in the simplest autoencoder. In an autoencoder, it uses an unsupervised way to eliminate the potential noise, which can retain the important information of the input data and simplify the output data and improve the effect of classification. A stacked autoencoder is stacked by multi-layer autoencoders. The previous autoencoder is the input of the next autoencoder, which forms the encoder and then adds the decoder part of the autoencoder to form an SAE with multiple hidden layers.

**FIGURE 4:**
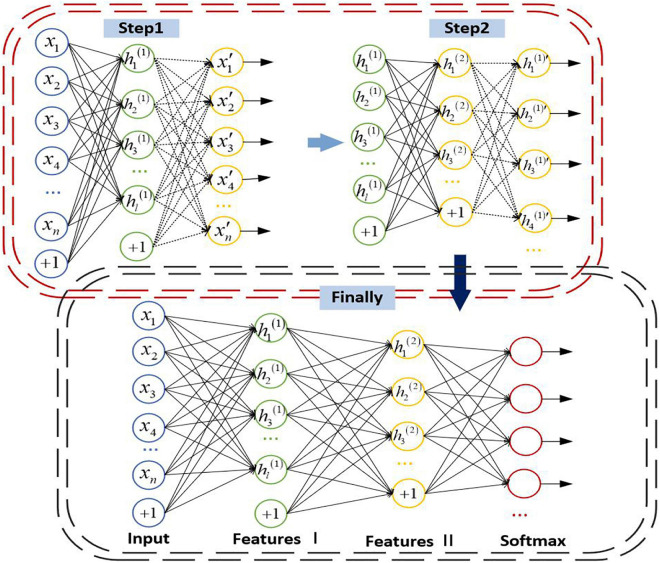
Structure of trestle autoencoder.

The parameters of the whole network are abundant since SAE contains numerous layers. It is prone to be overfitting if the end-to-end training method is adopted. To avoid overfitting, the autoencoder of each layer is trained individually from front to back, and only one hidden layer is trained each time. When the parameters of each layer are trained, the parameters of other layers remain unchanged. When the parameters are trained to convergence, the parameters of all layers are adjusted by the back propagation algorithm to improve the results. The details of the SAE are as follows: First, the sparse self-encoder network is used to train the parameters from the input layer to the H1 layer. After the training, the decoding layer is removed, leaving only the coding stage from the input layer to the hidden layer. Then we can train the parameters from the H1 layer to the H2 layer, and take the activation value of the H1 layer neurons without label data as the input layer of the H2 layer, and then conduct self-coding training. After training, we remove the decoding layer of H2, and so on. After training, you can connect the classifier SoftMax for classification tasks. The structure of SAE is shown in Figure 4.

The encoding steps of SAE are as follows:


(6) $$a^{\left(l\right)}=f\,\left(z^{\left(l\right)}\right)$$

(7) $$z^{\left(l+1\right)}=W^{\left(l,1\right)}\,a^{\left(l\right)}+b^{\left(l,1\right)}$$

The decoding steps are as follows:


(8) $$a^{\left(n+l\right)}=f\,\left(z^{\left(n+l\right)}\right)$$

(9) $$z^{\left(n+l+1\right)}=W^{\left(n-l,2\right)}\,a^{\left(n+l\right)}+b^{\left(n-l,2\right)}$$

where the activation value of the hidden layer unit can be denoted as *a*^(*n*)^. *W*^(*k*,1)^ is the parameter corresponding to *W*^(1)^, *W*^(*k*,2)^ is the parameter corresponding to *W*^(2)^, and *b*^(*k*,1)^ and *b*^(*k*,2)^ are the parameters corresponding to *b*^(1)^ and *b*^(2)^ in the *k*-th autoencoder, respectively. *n* is the number of neurons and *l* is the number of layers of the neural network.

The hidden layer of the last autoencoder is input to SoftMax as the feature of the input data for classification.

## The Proposed Emotion Recognition Algorithm

The algorithm mainly includes three steps: preprocessing the original EEG signals, feature extraction, and classification. The useless EEG signals in the DEAP dataset can be removed by using the filtering method due to doping different interference noise. Since then, the signal-to-noise ratio is improved to a certain extent, and the band is divided. EEG time series is decomposed into 5 bands (δ, θ, α, β, γ) through 3-order Butterworth bandpass filter (Wang et al., 2019), as shown in Table 1.

**TABLE 1:** Five bands of EEG.

EEG frequency band	Frequency range	Brain state	Level of consciousness
δ	0.5–4 Hz	Deep sleep	Very low
θ	4–8 Hz	Mild sleep	Low
α	8–12 Hz	Awake quiet, closed eyes, relaxed state	Medium
β	12–30 Hz	Mind active, focused, highly alert, anxious, excited	High
γ	30–100 Hz	Multimodal perception processing state, meditation	Very high

As the brain gradually wakes up, the frequency of EEG signals in the brain also gradually increases. In the δ band, the brain does not produce specific emotions in an unconscious state (sleep or coma), so we do not deal with this band in this paper. To remain the time-spatial feature, we use differential entropy to express the features of EEG signals. To improve the accuracy of recognition, we divide the 3 s baseline signal into three EEG segments with the length of 1 s, and each EEG signal segment is converted to 4 one-dimensional differential entropy feature vectors. Then the average value of these three feature vectors is taken as the baseline signal differential entropy feature of the subjects each time before they watched a specific emotional video. Finally, subjects under emotional video stimulation of brain electrical signals generated by the differential entropy features minus the baseline signal differential entropy to represent every emotional state feature of EEG segments.

The sensors that collect EEG signals are distributed in the brain, and it is intuitive to think that EEG signals from the same parts of the brain are fairly correlated. If the one-dimensional EEG signals are directly processed, the network will be forced to find the neighborhood correlation itself, which will affect the final recognition result. This neighborhood correlation can be obtained by using the representation of a two-dimensional matrix. When the one-dimensional EEG signals are mapped to the two-dimensional plane, the new data format can maintain the spatial information between adjacent electrode channels, which is conducive to mining the correlation between channels and is helpful to the convolutional autoencoder for emotion recognition.

**FIGURE 5:**
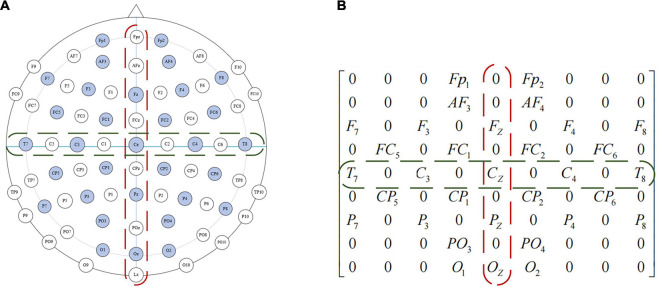
Electrode placement diagram and feature matrix mapping method of the international 10–20 system. **(A)** Electrode placement diagram. **(B)** Feature matrix mapping.

The location of the DEAP data collection device used in this paper is shown in Figure 5A, the EEG electrodes marked in light blue are the channels during the collection of DEAP dataset. To preserve the spatial features between EEG channels, we first turn the one-dimensional feature vector into a two-dimensional plane signal (size is*N*×*N*, where *N*is the maximum number of electrodes). For the DEAP dataset,*N*is 9, each of these channels can form a 9 × 9 matrix, such as in Figure 5B. If there is no electrode in the position, we assume that the gray value is 0.

In frequency dividing of the EEG signals, the frequency range of the four channels (θ, α, β, γ) has no overlap. Therefore, these four independent band signals can be used to encode EEG signals just like RGB images. In this paper, the four feature matrices formed by the four channels are stacked into a three-dimensional EEG signal cube to realize three-dimensional feature fusion, and the differential entropy feature in the EEG signals correspond to the gray intensity in the image. We encode the four independent bands of EEG signals, and the four feature matrices are stacked into a three-dimensional EEG signal cube, denoted as 3*D*_*cube* ∈ *R*^9×9×4^. Table 2 lists the terms corresponding to the representation of images and EEG signals.

**TABLE 2:** The term for images and EEG signals.

Image	EEG signal
Color image	Three-dimensional EEG signal cube
Color channel (R, G, B)	Signal frequency band (θ, α, β, γ)
Image grayscale value intensity	Differential entropy feature value

**FIGURE 6:**
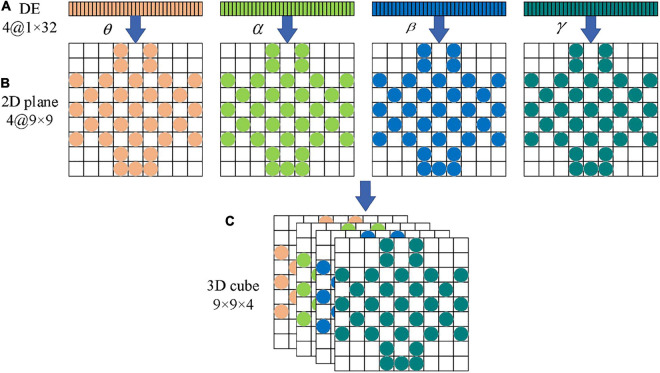
The flow chart of 32 channels one-dimensional EEG signal is transformed into a three-dimensional cube. **(A)** Electrode placement diagram. **(B)** Feature matrix mapping.

Figure 6 shows the overall flow chart of the 3D feature fusion. The steps are as follows: First, we divide the original EEG signals to four frequency bands. Second, we extract the differential entropy feature of each frequency. Third, we construct an image by inserting the pixel to the two-dimensional matrix. Finally, the two-dimensional features of each frequency band are fused to obtain a three-dimensional signal cube.

As can be seen from Figure 6, the three-dimensional EEG signal cube can be regarded as a color image with 4 primary color channels. Therefore, the ability of the CAE in feature extraction can be fully utilized, and high-dimensional features can be extracted effectively from the EEG signal cube. One hot encoder is used to code the feature in this paper.

The three-dimensional feature representation preserves the spatial correlation between EEG signal channels and integrates the differential entropy features of signals in different frequency bands. This feature representation can make the model achieve better recognition results. Before being input to the recognition model, the differential entropy features of all segments have been subtracted from differential entropy features of the corresponding baseline signals. After feature extraction, the input format of each sample data in convolutional autoencoder is an array of 9 × 9 × 4. The value at each position in the array represents the differential entropy feature value of the EEG signals with a length of 1 s at a particular frequency band at that position.

**FIGURE 7:**
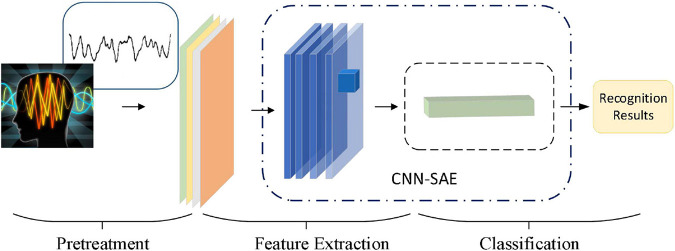
The network structure of the proposed algorithm.

In this paper, after obtaining 3D features, we construct the convolutional autoencoder to distinguish the state of emotion by combining CNN and SAE. Figure 7 shows the overall network structure.

Convolution neural network is a deep neural network with a core of convolution. To promote the recognition ability of CNN, we adopt the small scale of convolution kernels to extract local features in more detail and use four continuous convolutional layers to increase the non-linear expressiveness of the model. We also optimize the parameters of the network with dropout technology, and reduce the interdependence between neurons, and utilize L2 regularization to suppress network overfitting. The adaptive optimizer is used to optimize the model. Enter the output of the CNN network as a result of image feature extraction into the SAE. SAE is composed by a 2-layer sparse autoencoder. Batch normalization of input data on each sparse autoencoder is used to improve the accuracy of classification.

## Experiment

### Dataset

The DEAP dataset is a common public database for testing EEG emotion recognition algorithms. It includes 32 subjects, with 16 men and 16 women. Each subject watched 40 excerpts of music videos of 1-min duration of different emotional markers, with random video sequence and content, and the emotional excitation of each music video is looked at as a separate experiment. Multimedia stimulation materials combine visual and auditory ways to effectively induce emotions, which can effectively induce the emotional state of subjects. Before each subject watched the video, the testers collected baseline signals generated by a subject of 3 s without receiving an emotional stimulation state (relaxation state), and from each subject was collected 63 s of physiological signals, including a 3-s baseline signal and a 60-s experimental signal. The experiment collected subjects’ responses to the brain electrical signals of the music video and 8 surrounding physiological signals. The 8 peripheral physiological signals include EMG signals of musculus zygomaticus maximus and trapezius facialist, horizontal and vertical EOG signals, skin temperature, blood volume pulsation, and respiratory signals, basically covering several physiological signals commonly used in emotional recognition.

The brain electrical signals were collected at 512 Hz sampling frequency by using the “10–20” IAAF standard 32-guide electrode caps. All the subjects were required to fill in the self-evaluation form after watching the video, scoring from the continuous value range of 1–9 and marking the Valence and Arousal magnitude of the video watched. We use the 32-channel Biosemi active 2-device to record the brain electrical signals when subjects are exposed to the video. After the original data collection, the experimenter basically preprocessed the original data set and published the preprocessing version dataset for facilitating rapid verification by other researchers. All experiments in this paper are performed on the pretreatment version of the DEAP dataset. Table 3 shows the preprocessed dataset parameters.

**TABLE 3:** Overview of the preprocessing DEAP dataset.

Index	Parameter
Number of experiments	40 times/person
Experiment duration	60 s
Emotional division method	Two-dimensional mood model
Label type and label value interval	Valence, Arousal; continuous value 1–9
Signal acquisition frequency	128 Hz
Number of signal channels of different types	32 EEG channels

The DEAP dataset contains data and label. The experimental data format is shown in Table 4.

**TABLE 4:** Data styles of the subjects in the DEAP dataset.

Array name	Array dimension	Array content
Data	40 × 32 × 8,064	Video (number of experiments) × channels × sampled signal
Labels	40 × 2	Video (number of experiments) × label (valence, wake-up)

### Experimental Setting

In this paper, we focus on the emotion recognition with a short segment of the brain telecom signal. The original collected data with the length 8,064 by watching videos each time was divided into *n* segments with the length *l*, and all the *n* segments share the same emotion label. It is shown that for the DEAP dataset, 1 s is a more suitable time window length for emotion recognition (Yang et al., 2018b), so each 1-min brain signal is divided into 60 segments. Since the sampling frequency of the EEG acquisition device is 128 Hz, the 1-s EEG signal contains 128 signal points. For each subject, the total number of samples was 2,400.

To improve the recognition accuracy, we divide the 3-s baseline signal to three 1-s EEG segments, and transform each brain signal segment into four one-dimensional differential entropy feature vectors. We can gain the differential entropy feature of the baseline signal by averaging the three feature vectors of the subject before watching a specific emotional video. Eventually, the differential entropy features of the brain electrical signal are generated when the subject was stimulated by the emotional video, which minus the baseline signal’s differential entropy features represented the emotional state characteristics of each EEG segment. After several stages of data preprocessing, feature extraction, and feature fusion, the EEG signal storage format has changed differently, as shown in Table 5.

**TABLE 5:** Storage format of different processing states of EEG signals.

The EEG signal processing state	Storage format
Primary signal	40 × 8,064 × 32 (video × sampling signal × channel)
Frequency mode decomposition	40 × 8,064 × 4 × 32 (video × sampling signal × frequency band × channel)
Divide into short segment brain signal	40 × n × l × 4 × 32 (video × segment × segment length × frequency band × channel)
Feature extraction	40 × n × 4 × 32 (video × segment × frequency band × channel)
Feature fusion	9 × 9 × 4

**FIGURE 8:**
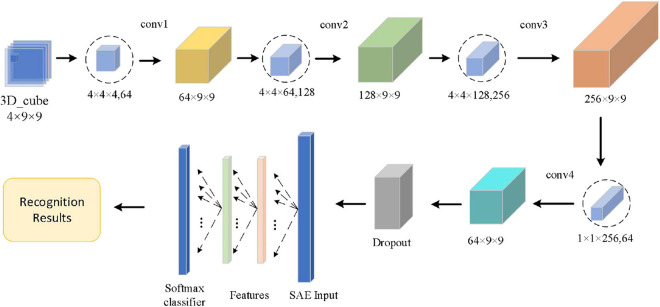
Network structure of the convolution autoencoder.

The convolutional autoencoder contains two parts, including convolutional layers and an autoencoder, which is shown in Figure 8.

As shown in Figure 8, the Conv1 layer is a convolution layer with an input by selecting the logical combination of one subject’s different bands in the arousal or valence. In Conv1, the number of convolution kernels is set to 64 and each convolution kernel’s size is 4 × 4 × 4. The Conv2 layer is a convolution layer, too. In Conv2, the size of the convolution kernel is 4 × 4 × 64 and the number of convolution kernels is set to 128. The Conv3 layer is also a convolution layer. In Conv3, the size of the convolution kernel is 4 × 4 × 128 and the number of convolution kernels is set to 256. To fuse the feature maps of the different channels and reduce the computational cost, the number of convolution kernels of the Conv4 layer is set to 64. In addition, in Conv4, the size of the convolution kernel is 1 × 1 × 256 and the stride size is 1. After four continuous convolutions, the brain signal cube is computed by the four times non-linear activation functions, which enhances the expression of the complexity of function and the non-linear degree and is beneficial to enhance the abstraction ability of local models. To preserve the edge information of the 3D EEG feature cube, we use a zero-padding operation in each convolution layer. In each convolution layer, the sizes of input and output are the same. Next to the convolution operation, we use ReLu activation function to realize the non-linear feature transformation of the model. In addition, the negative value is suppressed to 0 (namely, inactive state, positive feedback), which effectively solves the “gradient disappearance” problem. Since EEG data have features with high complexity and small sample, we use a dropout layer with a ratio of 0.2 to avoid over-fitting of the neural network. Finally, we send the output of the dropout layer into the SAE. The SAE network model totally has 4 layers—an input layer, two hidden layers, and an output layer. In the input layer, the number of neurons is the same as the dimension of the input sample. Moreover, in the two hidden layers, the number of neurons is set to 400 and 100, respectively. In the output layer, we use a SoftMax activation function to calculate the probability estimation of the emotional state. The SoftMax output layer is used to calculate the probability of category labels and output the results of emotion recognition.

The learning rate of the network can control the learning progress of the network model. There is a positive correlation between the speed of model training and the learning rate. But a larger learning rate can easily produce a loss value explosion and shock. The smaller learning rate may lead to overfitting and slow convergence. In this paper, we use Formula (12) to set the learning rate, that is,


(10) $$l\,e\,a\,r\,n\,i\,n\,g\,\_\,r\,a\,t\,e=\left\{ \begin{array}{cc} 10^{-4}, & t\,r\,a\,i\,n\,\_\,a\,c\,c\,u\,r\,a\,c\,y<0.7\\ 5\times 10^{-5}, & 0.7<t\,r\,a\,i\,n\,\_\,a\,c\,c\,u\,r\,a\,c\,y<0.85\\ 10^{-6}, & t\,r\,a\,i\,n\,\_\,a\,c\,c\,u\,r\,a\,c\,y>0.85 \end{array}\right.$$

### Experimental Results

Cross-validation (CV) is a tool of classification verification. In CV, we divide the overall dataset into a training set and a test set by a certain proportion. The training set is used for model training, and the test set is used for model testing. In our experiment, we adopt leave-out CV, fivefold CV, 10-fold CV, and 20-fold CV to verify the effect of sample size on classification accuracy. The following describes the experimental methods used.

Leave-out CV: We assume that the data set is (*x*_1_,*x*_2_,*x*_3_,⋯*x*_*n*_). The total number of samples is *n*. One sample is selected as the test set of the test stage, while the remaining *n*−1 samples are left as the training set of the training stage. After *n* times in turn, each sample has been a test set once, we can get the final classification accuracy by averaging all the test classification accuracy.

K-fold CV: We assume that the data set is (*x*_1_,*x*_2_,*x*_3_,⋯*x*_*n*_), the total number of samples is *n*. The data set is evenly divided into K groups. One group of the sample data is selected as the test set in the test stage, and the rest of the K-1 groups of sample data are selected as the training set in the training stage. The experiments are conducted K times in turn. Each group of sample data is regarded as a test set, and the classification accuracy is the average value of K classification results.

**FIGURE 9:**
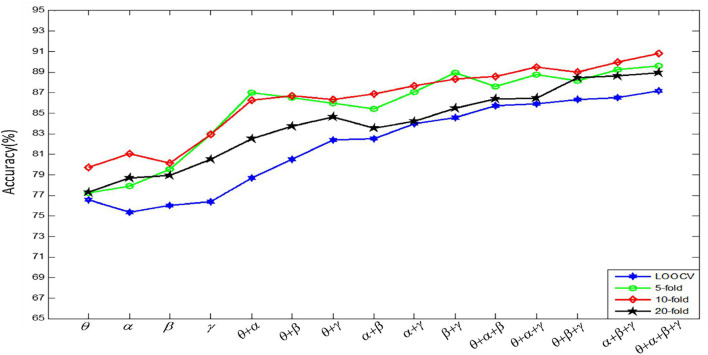
Four kinds of CV experiment results.

Figure 9 shows the results of emotion recognition by using a convolutional autoencoder for features constructed by different channels. Each recognition result is the recognition accuracy tested under four kinds of CV.

Figure 9 shows that the classification accuracy of K-fold CV is very high. However, the run time increases almost linearly with the increase of fold number. In order to balance the classification accuracy and run time, for each subject’s data, we use 10-fold CV to evaluate the recognition effect of the emotion recognition algorithm. In the follow-up experiments, we divide the data set into 10 parts, 1 of which is used as the test data, and the remaining 9 parts are used as the training data in turn. In our experiment, we randomly select 240 fragments as the test set, and the remaining 2,160 fragments as the training set. In addition, we can get the final recognition accuracy on the current subject data by averaging the recognition accuracy of the model on 10 test sets. Finally, the result, used to evaluate model accuracy, is the average of all the recognition accuracy of the model on the 32 subjects’ datasets.

To verify the validity of the constructed convolutional autoencoder, the proposed feature extraction algorithm of this paper is compared with CNN and MLP on several single rhythm signals and multi-band signals. The positive influence of a convolutional autoencoder on the classification result is verified. The network parameters of CNN are the same as the network parameter settings proposed in this paper. In CNN, the SAE structure is removed, and the recognition accuracy is obtained directly through the full connection layer output, which can show the influence of the SAE part of the proposed algorithm. In MLP, the input is a one-dimensional signal. The multilayer perception model introduces the ReLu activation function to avoid the disappearance of the gradient and improve the convergence speed. L2 regularization avoids the over-fitting problem and Adam optimizer optimizes the cross-entropy at different learning rates. The results of the experiment are given in Table 6.

**TABLE 6:** Comparison of model recognition accuracy.

Rhythm	Arousal			Valence		
	MLP	CNN	Our method	MLP	CNN	Our method
θ	70.68	78.09	**79.69**	68.70	75.66	**77.56**
α	67.70	80.81	**81.02**	61.97	78.73	**79.64**
β	69.43	79.97	**80.12**	65.90	78.13	**79.89**
γ	68.37	80.98	**82.90**	64.67	79.83	**80.19**
θ + α	80.88	85.30	**86.25**	80.06	83.60	**83.86**
θ + β	83.47	85.43	**86.65**	82.53	83.65	**84.05**
θ + γ	80.99	85.99	**86.29**	80.17	84.51	**84.93**
α + β	80.07	86.75	**86.83**	78.86	85.40	**85.62**
α + γ	80.21	87.55	**87.64**	78.07	86.77	**86.81**
β + γ	81.47	86.63	**88.28**	80.85	85.47	**86.51**
θ + α + β	86.79	88.45	**88.56**	85.32	87.34	**87.60**
θ + α + γ	85.86	89.12	**89.47**	84.92	87.89	**87.94**
θ + β + γ	86.85	88.55	**88.98**	86.15	87.50	**88.40**
α + β + γ	86.13	89.74	**89.91**	84.47	88.80	**88.95**
θ + α + β + γ	88.68	90.24	**90.76**	87.82	89.45	**89.96**

*The bold values are the best value in the according objective index.*

**FIGURE 10:**
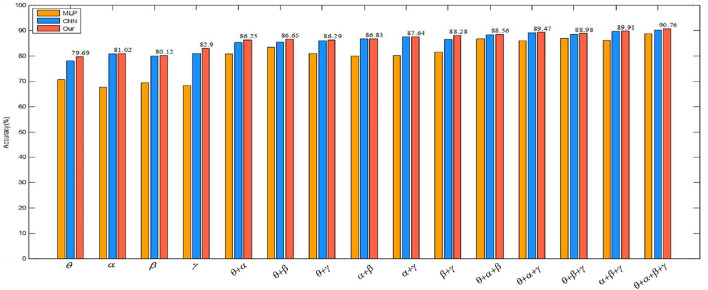
Comparison of recognition results of three methods in the arousal dimension.

To better illustrate the experimental results in Table 6, we visually compare the above results with the arousal of emotion recognition, as shown in Figure 10. The graph shows that the classification accuracy of the proposed algorithm is the highest, which shows the advantage of the convolutional autoencoder.

**FIGURE 11:**
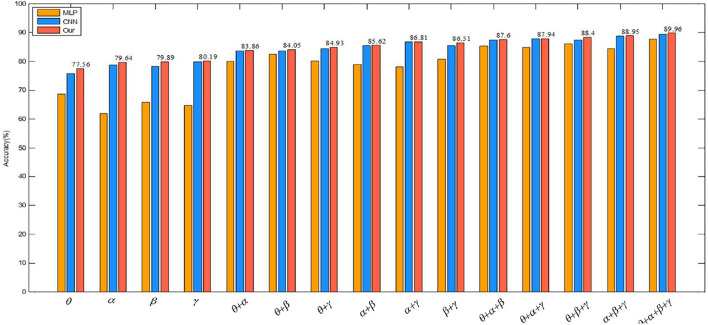
Comparison of three methods in the valence dimension.

We also visually compare the above results in the valence of emotion recognition in Figure 11. The graph shows that the classification accuracy of the proposed algorithm is also the highest, which also proves the effectiveness of the convolutional autoencoder.

**FIGURE 12:**
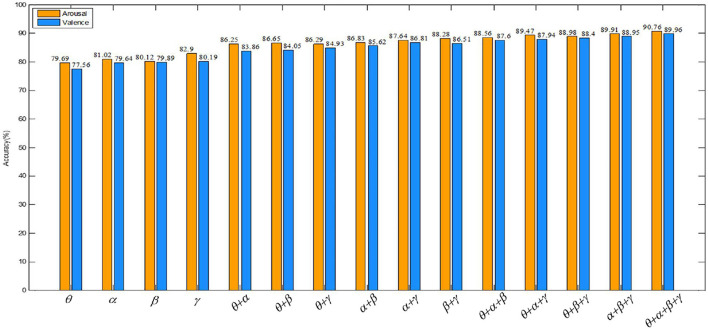
Comparison of the recognition accuracy of our method in the valence and arousal dimensions.

Valence describes the degree of an individual’s mood from negative to positive, distinguishing between positive and negative emotions. While arousal describes the degree of mood from calm to excitement and represents the degree of emotional arousal. In order to observe the comparison of the recognition accuracy of our algorithm in the valence and the arousal dimension more directly, a bar graph is drawn in Figure 12. Figure 12 shows that the recognition accuracy of the proposed algorithm is higher overall in the arousal dimension. The classification accuracy of our method in arousal is higher than in valence, which may be due to the influence of people’s subjective score when collecting EEG signals. In the same case, people are more likely to perceive their own emotional arousal.

To fully illustrate the effectiveness of our algorithm, we compared it with 9 other typical methods. These 9 algorithms include: (1) decision tree (DT) based emotion recognition algorithm presented in Reference (Liu et al., 2021); (2) support vector machine (SVM) based emotion recognition algorithm presented in Reference (Suykens and Vandewalle, 1999); (3) emotion recognition algorithm based on multi-layer perceptron (MLP) proposed in Reference (Yang et al., 2018a); (4) convolutional neural network (CNN) based emotion recognition algorithm proposed in Reference (Tripathi et al., 2017); (5) the emotion recognition algorithm based on the convolution recursive attention model (CRAM) presented in Reference (Zhang et al., 2019); (6) the multi-modal emotion recognition algorithm based on the Gauss process dynamics model (GPDM) presented in Reference (García et al., 2016); (7) emotion recognition algorithm based on phase space dynamics (PSD) presented in Reference (Soroush et al., 2019); (8) emotion recognition algorithm based on sparse discrimination ensemble (SDEL) presented in Reference (Ullah et al., 2019); and (9) emotion recognition algorithms based on continuous convolutional neural network (CCNN) proposed in Reference (Yang et al., 2018a). Table 7 shows the accuracy rates of all the algorithms in the valence and arousal dimensions, which proves that our algorithm is superior to other methods.

**TABLE 7:** Comparison results between the proposed algorithm and other algorithms.

Classification methods	DEAP dataset	
	Arousal (%)	Valence (%)
DT	71.16	68.28
SVM	87.43	86.60
MLP	88.88	87.73
CNN	73.40	81.40
CRAM	83.65	85.54
GPDM	90.60	88.30
PSD	87.42	84.59
SDEL	74.53	82.81
CCNN	90.42	89.45
Ours	**90.76**	**89.49**

*The bold values are the best value in the according objective index.*

As can be seen from Tables 6, 7, (1) high frequency band EEG is helpful to improve the accuracy of emotion recognition, which proves that emotion is produced in the waking state. (2) The combination of signal features of different frequency bands can complement each other, which can improve the accuracy of emotion recognition. (3) By comparing the multi-layer perceptron of one-dimensional input, the convolutional neural network of 3D input and the convolutional autoencoder of 3D input presented in this paper, it is found that the combination of 3D input data and convolutional autoencoder is more effective for emotion recognition. (4) A wider frequency band is helpful to improve the accuracy of emotion recognition because the single frequency band brings a bigger error to the experimental result.

## Conclusion

In this paper, we propose an EEG emotion recognition algorithm based on 3D feature fusion and convolutional autoencoder. In this paper, we used a Butterworth band-pass filter to divide the EEG signals to different frequency bands. The 3D features are constructed by using four frequency bands, and then the convolutional autoencoder is constructed as the emotion recognition classifier, which provides an efficient analysis method for emotion recognition. In this paper, through the experimental comparison of multi-layer perceptron, convolutional neural network, and the proposed convolutional autoencoder, we find that the constructed convolutional autoencoder has better performance in emotion recognition. We will further study how to retain the spatial characteristics of EEG signals as much as possible and strive to explore models and methods with shorter time and higher classification accuracy.

## Data Availability Statement

Publicly available datasets were analyzed in this study. This data can be found here: http://www.eecs.qmul.ac.uk/mmv/datasets/deap/.

## Author Contributions

YA performed the computer simulations and wrote the original draft. YA, YZ, and CX analyzed the data. SH and LZ revised and edited the manuscript. XD edited the manuscript. All authors confirmed the submitted version.

## Conflict of Interest

The authors declare that the research was conducted in the absence of any commercial or financial relationships that could be construed as a potential conflict of interest.

## Publisher’s Note

All claims expressed in this article are solely those of the authors and do not necessarily represent those of their affiliated organizations, or those of the publisher, the editors and the reviewers. Any product that may be evaluated in this article, or claim that may be made by its manufacturer, is not guaranteed or endorsed by the publisher.
